# A Case of COVID-19 Vaccine-Induced Thrombotic Thrombocytopenia

**DOI:** 10.7759/cureus.27204

**Published:** 2022-07-24

**Authors:** Hailey Harrison, Hadi Rezaei, Nimit Dalal

**Affiliations:** 1 Internal Medicine, American University of Antigua, New York City, USA; 2 General Surgery, Mayo Clinic, Rochester, USA; 3 Internal Medicine, Trumbull Regional Medical Center, Warren, USA

**Keywords:** venous and arterial thrombosis, heparin induced thrombocytopenia (hit), vitt covid-19, vaccine-induced thrombotic thrombocytopenia (vitt), covid 19

## Abstract

This report discusses a case of a 37-year-old female who developed vaccine-induced thrombotic thrombocytopenia (VITT) after receiving the Johnson and Johnson COVID-19 vaccination. The patient first presented to the ED with complaints of a worsening headache. Labs were significant for thrombocytopenia with a platelet count of 22,000, and the patient was admitted to the inpatient unit for monitoring. The day after admission, the patient was found to have a right common femoral artery embolus, left distal popliteal trifurcation embolism, a small pulmonary embolism in the right lower lobe, and a mural thrombus of the infrarenal abdominal aorta. Following these findings, the patient underwent emergent thrombectomy of the common and superficial femoral arteries. Over the hospital course of six days, the patient received steroids and IV immunoglobulin (IVIG), which led to the resolution of the thrombocytopenia. The patient was given argatroban followed by apixaban for anticoagulation. She was instructed to follow up with hematology within one to two weeks post-discharge for monitoring of anticoagulation and thrombus surveillance. This case report outlines the clinical course, diagnosis, and treatment of a case of VITT, which will assist physicians in early recognition and adequate treatment of this condition as the COVID-19 pandemic continues.

## Introduction

Few serious side effects have been reported from the administration of the various new vaccines that were developed during the coronavirus pandemic [1]. However, vaccine-induced thrombotic thrombocytopenia (VITT) is a very rare prothrombotic syndrome that has been reported in some patients after receiving coronavirus vaccination with the adenovirus vector-based vaccines: AstraZeneca (ChAdOx1 nCoV-19) and Johnson and Johnson (Ad26.COV2.S) [2].

VITT is a thrombotic syndrome that involves the development of immunoglobulin G (IgG) antibodies that bind to the Fc portion of the IgG receptor on platelet factor-4 (PF4). Platelet activation occurs upon binding, a phenomenon similar to heparin-induced thrombocytopenia (HIT). The immunopathology of VITT differs from HIT since the antibodies bind to a different epitope on PF4, and VITT is not dependent on exposure to heparin products. The mechanism in which VITT causes antibody formation is still unclear. Some theories suggest vaccine components may generate a neoantigen when bound to PF4 [2]. VITT is a very rare complication of the adenoviral-vector-based COVID-19 vaccines, with the CDC estimating an incidence of 1 in 533,333 [3]. In this report, we present a case of VITT in an adult female with no previous medical issues.

## Case presentation

A 37-year-old female with no previous health conditions presented to the ED with an 11-day history of headaches following vaccination with the Johnson and Johnson COVID-19 adenovirus-based vaccine. The headache was localized to the bi-temporal region, was constant in nature, and had been progressively worsening since the time of vaccination. The patient had a past surgical history of cholecystectomy and Cesarean section. She had no known medical conditions and was a current cigarette smoker with a 10-pack-year history. The patient reported a positive history of blood clots in her sister and mother, with no known diagnoses of hereditary coagulopathies. Vital signs on arrival were within the normal limits: temperature of 98.1℉, heart rate of 72 beats per minute, blood pressure of 114/78 mmHg, and oxygen saturation of 96% on room air. Labs in the ED were significant for thrombocytopenia, with a platelet count of 22,000. Other pertinent lab values include an elevated C-reactive protein (CRP) of 3.19 mg/dL. β-HCG was negative, and urinalysis showed no evidence of urinary tract infection. At the time of presentation, the patient had no signs of active internal bleeding, petechiae, purpura, or ecchymosis. Physical examination was unremarkable, although the patient appeared to be in significant distress because of the headache. A non-contrast CT scan of the head was ordered and showed no acute abnormalities. A chest X-ray was also performed and demonstrated no acute cardiopulmonary disease. The COVID-19 rapid antigen test was positive, and a polymerase chain reaction (PCR) test was ordered. A full respiratory serology panel was performed and was negative. The patient received a 500 mL bolus of sodium chloride, 30 mg of IV ketorolac, and 10 mg of IV dexamethasone and was admitted to the general medical floor for observation and management of thrombocytopenia.

**Figure 1:**
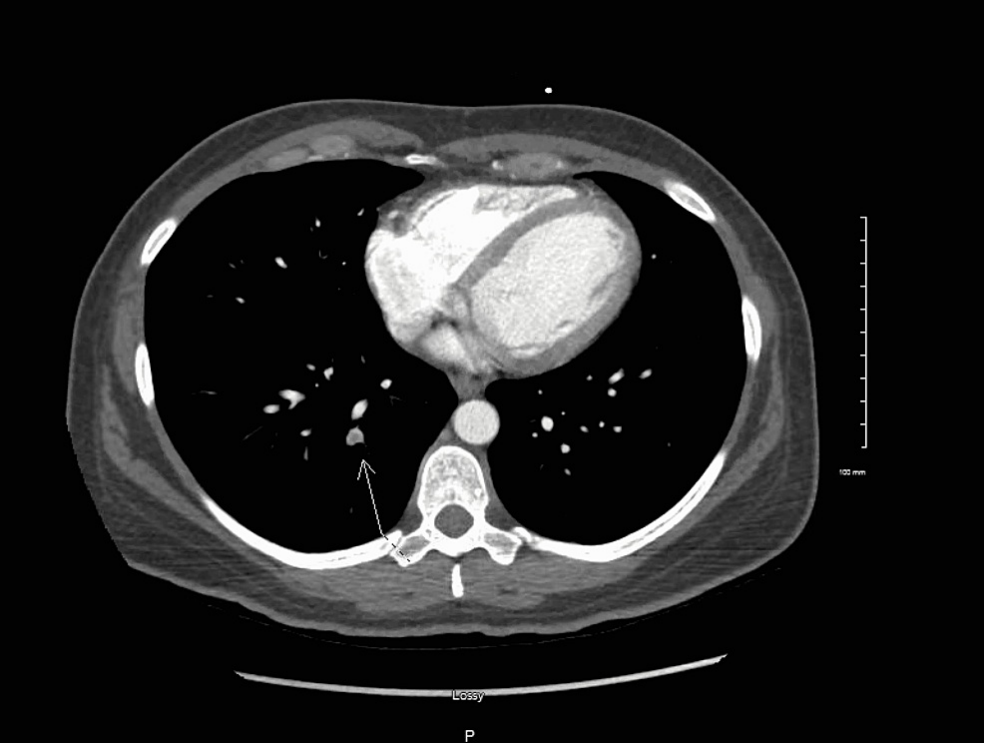
Small pulmonary embolism in the right lower lobe.

**Figure 2:**
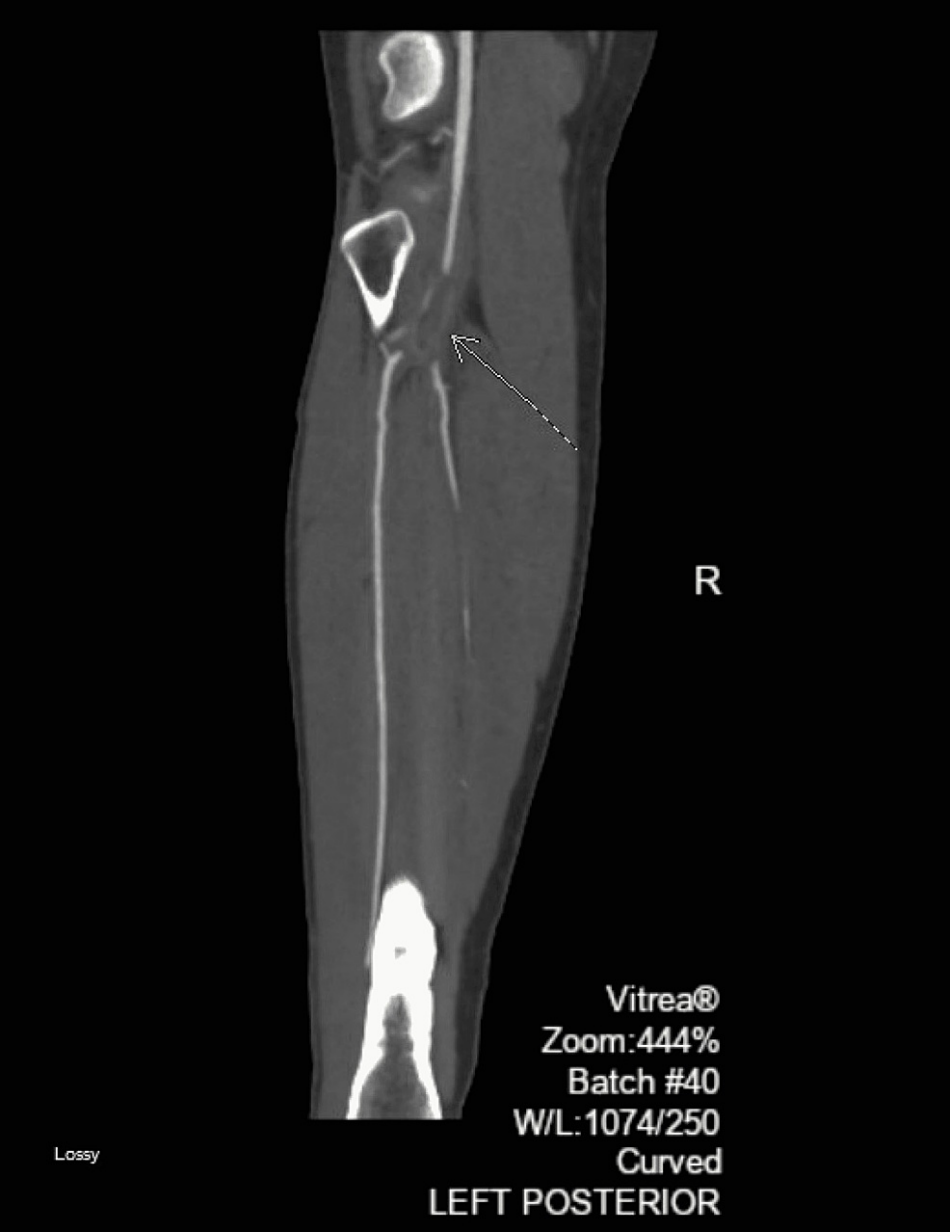
Left distal popliteal trifurcation embolus with segmental occlusion.

**Figure 3:**
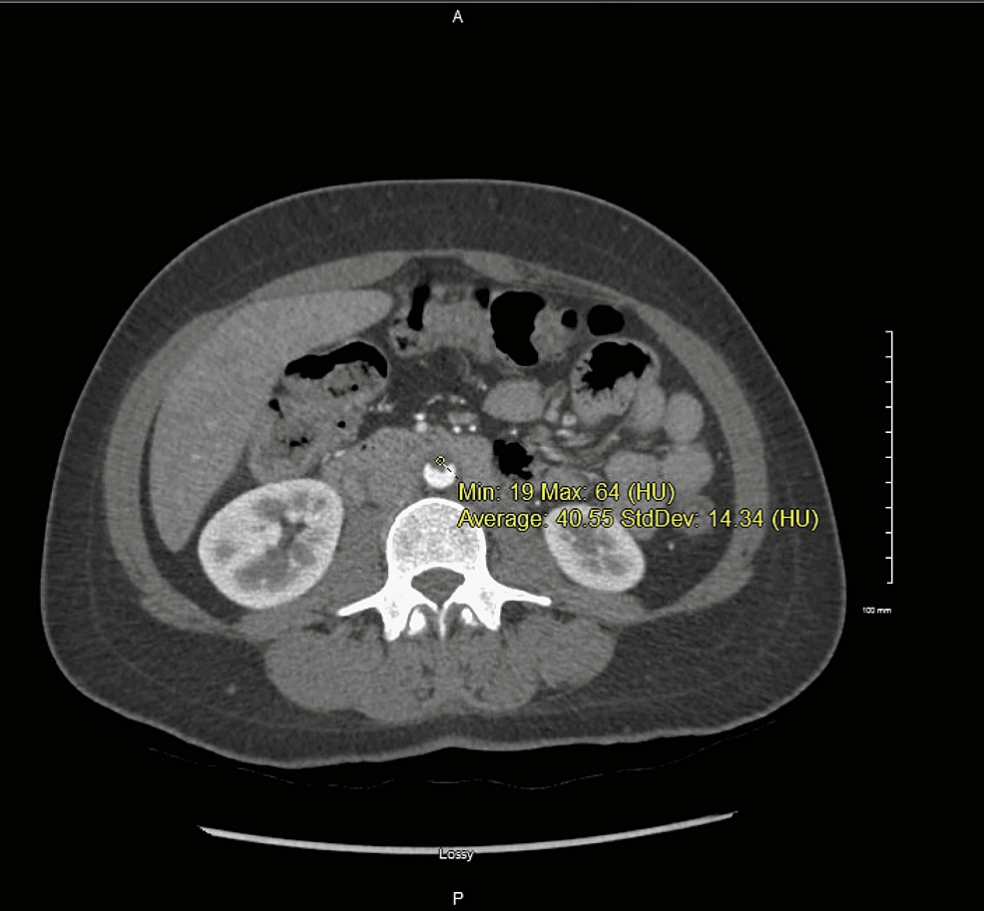
Mural thrombus or soft plaque on the anterior wall of the infrarenal abdominal aorta.

On day 1 of hospitalization, the patient woke up with sudden excruciating right lower extremity pain. At this time, she was diaphoretic, tachycardic, and flushed. The pain was associated with numbness, tingling, and decreased sensation around the right ankle with preserved motor function. The dorsal pedal, posterior tibial, and popliteal pulses were non-palpable and non-Dopplerable. Labs showed a high d-dimer level of 6.01 (normal 0.19-0.5), a low platelet count of 20,000, and an elevated immature platelet fraction of 14.3%. Fibrinogen was normal at 217, and coagulation studies, including prothrombin time (PT), partial thromboplastin time (PTT), and International Normalized Ratio (INR), were within the normal limits. CRP was elevated at 2.67, and procalcitonin was normal at 0.06 ng/mL. Venous duplex ultrasound of the right lower extremity showed no evidence of deep vein thrombosis. A computed tomography angiography (CTA) scan of the abdominal aorta with runoff was ordered and showed a right common femoral artery embolus extending into the origin of the superficial femoral and profunda artery, a left distal popliteal trifurcation embolism with segmental occlusion, a small pulmonary embolism in the right lower lobe, and a mural thrombus of soft plaque in the anterior wall of the infrarenal abdominal aorta (Figures 1-3). The patient was not a candidate for thrombolysis due to thrombocytopenia, so an open thrombectomy of the right common and superficial femoral arteries was done with an embolectomy catheter. Post-operatively, the patient received anticoagulation with argatroban. 

Following the procedure, the patient’s platelet count had decreased to 12,000, the WBC was elevated at 12,400, hemoglobin (Hb) was low at 11.6, and PTT was increased to 39.9. She was admitted to the ICU and was started on 10 mg IV dexamethasone Q6H for four days. The COVID-19 PCR test came back negative, confirming a false-positive rapid antigen test. Pathology was consulted to examine a peripheral blood smear, which showed normal platelet morphology with a decrease in the number of platelets. The pathologist suggested that this may be secondary to peripheral consumption and SARS-CoV-2 vaccine-induced immune thrombotic thrombocytopenia. The patient’s serum was tested for heparin-platelet factor 4 (heparin-PF4) antibodies. Heparin-PF4 antibody enzyme-linked immunosorbent assay (ELISA) was positive with 100% heparin-PF4 antibody inhibition and an optical density of 2.265. Hematology was consulted and suggested that thrombosis is related to a HIT-like mechanism, even without known exposure to heparin products. This was concluded by cases in the literature that showed immune-induced thrombocytopenia with vascular thrombotic events after COVID-19 vaccination, mostly after receiving the AstraZeneca vaccine. It was recommended that low-weight molecular heparin and platelet transfusions should be avoided to prevent worsening thrombosis. Hematology requested that the patient be started on IV immunoglobulin (IVIG) for two days if there was worsening thrombocytopenia or any signs of bleeding.

On hospital day two, morning labs showed that the platelet count dropped to 8000. The patient received her first day of IVIG. WBC count at this time increased to 14,300. Immature platelet fraction had increased to 19.6%, and PTT increased to 41.4. At this time, hematology suggested that platelet transfusions should be withheld unless active bleeding is present. Venous duplex ultrasound of the unaffected left leg was taken and showed no evidence of deep vein thrombosis (DVT). Venous duplex ultrasounds of the bilateral upper extremities showed acute DVT in the left brachial vein. MRI of the brain showed no evidence of acute infarct, parenchymal hemorrhage, cerebral edema, or cerebellar tonsillar ectopia. Magnetic resonance angiogram (MRA) showed no significant stenosis of the major intracranial arteries and did not identify any medium or large-sized aneurysms. Magnetic resonance venography (MRV) showed no evidence of dural venous thrombosis. CT scan of the brain and head with contrast showed no evidence of dural venous sinus or cavernous sinus thrombosis and no acute intracranial hemorrhage or mass effect. Repeat afternoon labs showed some improvement in platelet count, rising to 14,000. WBC count at this time continued to trend upward to 16,200. Immature platelet fraction increased to 21.6%. Coagulation studies showed prolonged PT of 16.3 and PTT of 45. Other lab abnormalities include low fibrinogen of 135 and an elevated aspartate aminotransferase (AST) of 123.

On hospital day three, the platelet count continued to trend upwards to 20,000, WBC count remained stable at 14,500, Hb was low at 10.3, immature platelet fraction remained stable at 22.3%, PTT remained stable at 44.5, and AST was high but trending downwards to 105. The head CT scan showed no evidence of dural venous sinus or cavernous sinus thrombosis and no acute intracranial hemorrhage. The patient received day two of IVIG. 

On hospital day four, the platelet count increased to 47,000. The WBC count trended downwards to 13,100. Hb remained low but stable at 10, PT remained elevated but stable at 24.3, PTT was high but stable at 50, and AST continued to trend down to 69.

On hospital day five, the platelet count improved to 101,000. WBCs trended down to 12,200, Hb was 10.4, PTT was 48.4, and AST was 42, trending towards normal. The patient was discontinued on argatroban and started on oral apixaban 10 mg two times a day (BID) every 12 hours (Q12H) for seven days. The dexamethasone changed from IV to 10 mg per os (PO) today, and she was transferred from the ICU to the medical floor. Pathology confirmed that the content analyzed from the femoral arteries was consistent with a thrombus. 

On hospital day six, the platelet count improved to 143,000. WBC count was elevated at 17,500, Hb increased to 11.7, and AST trended down to 52. Dexamethasone was tapered to 10 mg BID, then discontinued upon discharge. The patient was told to decrease the apixaban dose to 5 mg BID after seven days.

Lab values recorded during the hospital stay are summarized in Table 1.

**Table 1:** Pertinent lab values over hospital stay. CRP: C-reactive protein; PTT: Partial thromboplastin time; PT: Prothrombin time; AST: Aspartate aminotransferase.

Lab value (Reference range)	Hospital Day 0 (admission)	Hospital Day 1 (preop)	Hospital Day 1 (postop)	Hospital Day 2 (am)	Hospital Day 2 (pm)	Hospital Day 3	Day 4	Day 5	Day 6
Platelet count (150,000-450,000)	22,000	20,000	12,000	8000	14,000	20,000	47,000	101,000	143,000
CRP (<0.3 mg/dL)	3.19	2.67							
Immature platelet fraction (0.9-11.2%)		14.3		19.6	21.6	22.3			
WBC (3500-10,500)	6400		12,400	14,300	16,200	14,500	13,100	12,200	17,500
Hemoglobin (12-15.5 gm/dL)	12.1	12.7	11.6	10.7	10.2	10.3	10	10.4	11.7
PTT (22.3-32.8 seconds)		25.3	39.9	41.4	45.1	44.5	50	48.4	
Fibrinogen (mg/dL)		217			135				
PT (9.5-13.5 seconds)		11.2			16.3	24.3			
AST (10-37 unit/L)		7			123	105	69	42	52

## Discussion

VITT is an uncommon but potentially life-threatening complication that has developed after vaccination with AstraZeneca and Johnson and Johnson COVID-19 adenoviral-based vaccines. This led to safety concerns as several individuals received these vaccinations during the COVID-19 pandemic. However, studies reveal that there is no association between the development of VITT and the BioNTech Pfizer vaccine [4]. One case report has also been published on fatal thrombotic events following vaccination with Moderna. However, it is unknown if this was a direct result of VITT or was related to a prior illness [5]. We reported a case of VITT following the Janssen (Johnson and Johnson) COVID-19 vaccination. A case report in the UK that included 220 cases of definite or probable VITT stated that the median time of diagnosis post-vaccination is 14 days, ranging from 5 to 48 days [6]. This study showed a female predominance among cases, with 55% of patients being female, making this a significant risk factor for VITT [6]. Over half of the patients studied had multiple thrombi present in different locations. The patient in our case report had similar demographics to this study as she presented to the hospital with symptoms 11 days post-vaccination, is female in gender, and had four different thrombotic sites. In systematic reviews, the headache was shown to be the most common presenting symptom in patients with VITT and cerebral venous sinus thrombosis (CVST) [7]. Despite our patient’s presentation of an unremitting headache, no signs of CVST or neurological abnormalities were found on imaging.

Sites of venous thrombosis in VITT can vary but are more commonly seen as deep vein thrombosis of the lower extremities and thromboembolism to the lungs [1,8]. Some rare but observed sites of thrombosis in VITT include the splenic, portal, mesenteric, adrenal, cerebral, and ophthalmic veins [1,8]. CVST is an uncommon but serious complication of VITT, where thrombosis of the cerebral sinuses occurs, leading to intracranial hypertension [9]. Thrombosis in the presence of VITT can be diagnosed through MRI with venography or CT venography. This was done after our patient developed thrombosis in the setting of thrombocytopenia, thus ruling out dural sinus thrombosis as a cause of the headache [1,9].

Arterial thrombosis caused by VITT has been displayed through reports of middle cerebral artery stroke and occlusion of peripheral arteries [10]. Our patient’s sites of thrombosis are consistent with these locations as they include the femoral arteries, popliteal artery, pulmonary vasculature, and abdominal aorta. The median platelet count for patients with VITT is 20,000-25,000 [2]. In this report, the patient’s platelet count dropped as low as 8000 but mostly remained within the 10,000-20,000 range. VITT is diagnosed by using PF4 antibody tests [11]. A diagnosis is based on a positive PF4 antibody assay and the presence of thrombocytopenia or thrombosis. The test used on our patient was the ELISA, which is the recommended screening test [11]. Other screening tests include serotonin release assay, which can be used in patients with suspected VITT with a negative or equivocal ELISA [12]. Patients diagnosed with VITT from case reports in the UK had high optical densities on ELISA, ranging between a density of 2 and 3 [2]. The patient in our report had an optical density of 2.265. Our patient was treated for two days with IVIG infusions, one of the mainstays of treatment in VITT, as it substantially improves the patient’s platelet count while stabilizing coagulative events [13]. IVIG interferes with the ability of PF4 to activate platelets by blocking FcRyƳIIA receptors, as seen in HIT [14]. Plasma exchange is another potential treatment that temporarily reduces the PF4 antibodies, thus decreasing coagulability [7]. It is important to note that platelet infusions should be avoided in the case of VITT to prevent further antibody formation and thrombosis [14]. The long-term complications of PF4 antibodies have not yet been established [13]. An observational study showed that VITT patients had a negative platelet functional assay within a median time of 15.5 weeks [15]. However, 7.5% of subjects showed persistently high levels of antibodies and optical densities, and two out of these five patients had a recurrent episode of thrombocytopenia [15]. Due to the new emergence of this condition, the prolonged implications of VITT and PF4 antibodies will be an essential topic for research in the coming years.

## Conclusions

Our patient presented with a case of vaccine-induced thrombotic thrombocytopenia, a recent illness that arose with the development of vaccines produced in light of the COVID-19 pandemic. VITT can be life-threatening if not recognized quickly and treated adequately. In this case, the patient was treated surgically and medically for multiple thrombotic events in the presence of thrombocytopenia, leading to a full recovery prior to discharge. VITT is a very rare complication of the adenoviral-vector-based COVID-19 vaccines, and although it can result in serious medical issues, the benefits of protection against COVID-19 heavily outweigh any associated risks.
